# Designing a Comprehensive Family Caregiver Assessment: Insights from a Multidisciplinary Professional Panel

**DOI:** 10.1002/alz.092990

**Published:** 2025-01-09

**Authors:** Shadi Gholizadeh, Matthew Neal, Kirk Hayes, John Mark Geiss

**Affiliations:** ^1^ Brigade Health, Orange, CA USA; ^2^ Senior Doc, Orange, CA USA

## Abstract

**Background:**

The imminent launch of the Guiding an Improved Dementia Experience (GUIDE) Model necessitates a nuanced understanding of family caregiver needs to support the implementation of family‐centered dementia care plans. Recognizing the multifaceted role of family caregivers, a preliminary working session was convened to outline the foundational elements of a family caregiver assessment, ensuring these assessments are equipped to guide effective interventions.

**Method:**

A multidisciplinary panel consisting of a clinical psychologist, two health care executives, and a physician with geriatric expertise convened to delineate components of a caregiver assessment tool to supplement the Zarit Burden Interview. The panel, representing diverse expertise within the elder care setting, engaged in discussions focused on the challenges caregivers face, the support they require, and the pivotal information that could enhance care delivery. The session aimed to consolidate professional insights in order to develop a focus group interview guide as a precursor to engaging with the broader caregiver community.

**Result:**

The panel identified essential domains for the caregiver assessment, including caregiver’s understanding of dementia, behavioral modification strategies, navigation of healthcare systems, and caregiver health behaviors. It was determined that each of these domains should be developed after literature review and insights from family caregivers. Five areas that were highlighted as often missed but crucial to include in an assessment were 1) the caregiver’s understanding of anosognosia as a dementia symptom, 2) a plan and goal setting for what the caregiver would do during respite times, 3) caregiver health behaviors, 4) cultural considerations around caregiver roles and identity and 5) learning preferences. Failing to consider language/cultural preferences and preferred modalities of support modalities emerged as a common barrier to caregiver engagement, affecting care plan acceptance.

**Conclusion:**

Recognizing the preliminary nature of this professional panel’s insights, the next phase will involve conducting extensive focus groups with family caregivers to validate and expand upon these findings. This subsequent step is pivotal in ensuring the assessment tool captures the authentic voice, needs, and preferences of caregivers, thereby facilitating the delivery of comprehensive, person and family‐centered dementia care.

